# Solubilization and Thermodynamic Analysis of Isotretinoin in Eleven Different Green Solvents at Different Temperatures

**DOI:** 10.3390/ma15228274

**Published:** 2022-11-21

**Authors:** Faiyaz Shakeel, Nazrul Haq, Wael A. Mahdi, Ibrahim A. Alsarra, Sultan Alshehri, Miteb Alenazi, Abdulrahman Alwhaibi

**Affiliations:** 1 Department of Pharmaceutics, College of Pharmacy, King Saud University, Riyadh 11451, Saudi Arabia; 2 Department of Clinical Pharmacy, College of Pharmacy, King Saud University, Riyadh 11451, Saudi Arabia

**Keywords:** correlation, isotretinoin, solubility, Hansen solubility parameter, thermodynamic analysis

## Abstract

The solubilization and thermodynamic analysis of isotretinoin (ITN) in eleven distinct green solvents, such as water, methyl alcohol (MeOH), ethyl alcohol (EtOH), 1-butyl alcohol (1-BuOH), 2-butyl alcohol (2-BuOH), ethane-1,2-diol (EG), propane-1,2-diol (PG), polyethylene glycol-400 (PEG-400), ethyl acetate (EA), Transcutol-HP (THP), and dimethyl sulfoxide (DMSO) was studied at several temperatures and a fixed atmospheric pressure. The equilibrium approach was used to measure the solubility of ITN, and the Apelblat, van’t Hoff, and Buchowski–Ksiazczak *λh* models were used to correlate the results. The overall uncertainties were less than 5.0% for all the models examined. The highest ITN mole fraction solubility was achieved as 1.01 × 10^−1^ in DMSO at 318.2 K; however, the least was achieved as 3.16 × 10^−7^ in water at 298.2 K. ITN solubility was found to be enhanced with an increase in temperature and the order in which it was soluble in several green solvents at 318.2 K was as follows: DMSO (1.01 × 10^−1^) > EA (1.73 × 10^−2^) > PEG-400 (1.66 × 10^−2^) > THP (1.59 × 10^−2^) > 2-BuOH (6.32 × 10^−3^) > 1-BuOH (5.88 × 10^−3^) > PG (4.83 × 10^−3^) > EtOH (3.51 × 10^−3^) > EG (3.49 × 10^−3^) > MeOH (2.10 × 10^−3^) > water (1.38 × 10^−6^). ITN–DMSO showed the strongest solute–solvent interactions when compared to the other ITN and green solvent combinations. According to thermodynamic studies, ITN dissolution was endothermic and entropy-driven in all of the green solvents tested. The obtained outcomes suggested that DMSO appears to be the best green solvent for ITN solubilization.

## 1. Introduction

**Figure 1:**
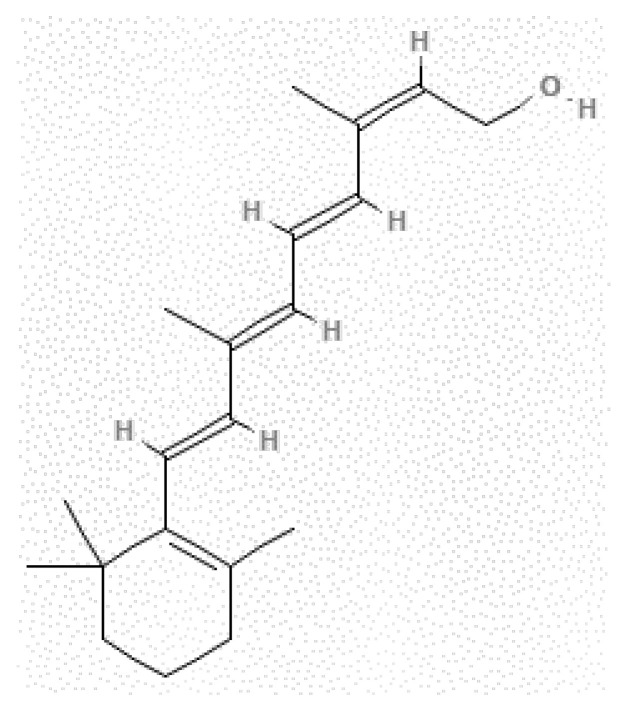
Chemical structure of isotretinoin (ITN).

Isotretinoin (ITN) (chemical structure: Figure 1; IUPAC name: (2*Z*,4*E*,6*E*,8*E*)-3,7-dimethyl-9-(2,6,6-trimethylcyclohexen-1-yl)nona-2,4,6,8-tetraenoic acid; molecular formula: C_20_H_28_O_2_; molar mass: 300.44 g mol^−1^, PubChem ID: 5282379, and CAS number: 4759-48-2) is commercially available as yellow-orange to orange crystalline powder [1,2]. It is a *cis* configurational isomer of retinoic acid, commonly known as 13-*cis*-retenoic acid [1,3]. Since ITN plays an important role in controlling gene expression, it was found to be suitable for the treatment of various cancers [4,5]. It was also found to be useful in treating various skin diseases such as acne, psoriasis, and skin carcinoma [6,7,8,9,10]. It is claimed to be the best medication for the treatment of acne [10,11]. Recently, a practical method for the oral administration of ITN in pediatric neuroblastoma patients was discovered as the patient was not able to swallow commercially available tablets or capsules [12].

ITN is a highly lipophilic compound and possesses poor solubility in water, which results in difficulties in its formulation development [13,14]. It is uncommon to find information about ITN’s solubility and other physicochemical properties in the literature. Industries have a larger need for solubility data, solubility parameters, and other physicochemical data of weakly water-soluble compounds in aqueous and organic solvents [15,16,17,18]. Some lipid-based formulations such as microemulsions, microemulsion gels, and self-nanoemulsifying formulations were examined to alter the physicochemical and fundamental properties of ITN [19,20,21,22,23,24]. At room temperature, the solubility of ITN was reported in certain green solvents, including propane-1,2-diol (PG), polyethylene glycol-400 (PEG-400), and Transcutol-P (THP) [20,23].

However, the temperature-dependent solubility data and thermodynamic characteristics of ITN in examined environmentally friendly/green solvents, including water, methyl alcohol (MeOH), ethyl alcohol (EtOH), 1-butyl alcohol (1-BuOH), 2-butyl alcohol (2-BuOH), ethane-1,2-diol (EG), PG, PEG-400, ethyl acetate (EA), THP, and DMSO have not yet been published. Therefore, new solubility data and thermodynamic parameters for ITN in eleven different environmentally friendly solvents—water, MeOH, EtOH, 1-BuOH, 2-BuOH, EG, PG, PEG-400, EA, THP, and DMSO—at a fixed atmospheric pressure (101.1 kPa) and at temperatures ranging from 298.2 to 318.2 K are reported in the current work. To assess the way ITN dissolves in various green solvents, “apparent thermodynamic analysis” was used. The solubility and physicochemical data found in this study may be useful for the purification, recrystallization, drug discovery, pre-formulation evaluation, and dosage form design of ITN.

## 2. Materials and Methods

### 2.1. Materials

ITN working compound was provided by BOC Sciences (Shirley, NY, USA). MeOH, EtOH, 1-BuOH, 2-BuOH, EG, PG, and PEG-400 were procured from Fisher Chemical (Loughborough, UK). EA and DMSO were acquired from Sigma Aldrich (Darmstadt, Germany). THP was obtained from Gattefosse (Lyon, France). The Milli-Q unit was used to obtain ultra-pure water. Supplementary Table S1 provides an overview of each material’s specifics.

### 2.2. Analytical Methodology for Determination of ITN

A laboratory-developed and validated high-performance liquid chromatography (HPLC) approach was utilized to determine ITN in all in vitro samples. ITN analysis was carried out using a UV–Vis detector at a wavelength of 354 nm. ITN was subjected to the analysis using an HPLC system (Waters, Milford, MA, USA) at room temperature (298.2 K). For the HPLC determination of ITN, a Nucleodur (150 × 4.6 mm, 5 μm) RP C_18_ column was utilized. The green solvent mixture of ethyl alcohol and ethyl acetate (50:50, *v/v*) flowed at a flow speed of 1.0 mL min^−1^. The green solvent mixture was freshly made, filtered through a 0.45 µm nylon filter, and then degassed before analysis. A 20 µL injection volume was used. By plotting ITN concentrations against the measured HPLC area, the ITN calibration plot was created. The ITN calibration curve was linear between 0.2 and 80 µg g^−1^, with a determination coefficient (R^2^) of 0.9994. ITN has a regression line equation of *y* = 75,179*x* + 59,230, where x and y are the measured HPLC area and ITN concentration, respectively.

### 2.3. Characterization of Solid Phases of ITN

The solid phases of pure ITN and equilibrated ITN (the sample obtained from the equilibrated MeOH) were characterized using differential scanning calorimetry (DSC), Fourier transforms infrared (FTIR) spectroscopy, and X-ray diffraction (XRD) studies. The equilibrated ITN was recovered from MeOH via slow evaporation [17,18]. DSC measurements were performed using a DSC-60 Instrument (Shimadzu, Tokyo, Japan) to obtain DSC spectra for both samples. Pure indium was used as the calibration reference and the apparatus was calibrated between 283.2 K and 773.2 K. Aluminum pans were used to hold the precisely measured 4.0 mg of pure and equilibrated ITN that were hermetically sealed with the aid of aluminum lids and a crimper. An empty aluminum pan was used as a reference for the thermal scanning of both pure and equilibrated ITN. Both samples’ DSC spectra were captured at a nitrogen flow rate of 40 mL min^−1^. For each sample, the heating rate was set to 10 K min^−1^. Both samples were heated to a temperature between 298.2 and 523.2 K. Using TA-60WS thermal analysis software (Shimadzu, Tokyo, Japan), various thermal parameters for both samples were calculated [25].

Utilizing the KBr disc method as described in the literature [26], the absorption spectra in the range of 300 cm^–1^–4000 cm^–1^ were acquired for FTIR spectral analyses. The samples were examined using a Diffractometer (Rigaku Inc. Tokyo, Japan) fitted with Cu-K radiation of 1.5406 for XRD measurements. In the 2–60° diffraction angle (2θ) range with a 0.02° step size, both ITN samples were investigated [18]. The potential conversions of ITN into different physical states, including polymorphs, solvates, and hydrates, among others, were investigated using the DSC, FTIR, and XRD investigations.

### 2.4. ITN Solubility Determination

At different temperatures and fixed pressure, the solubility of ITN in eleven different green solvents was determined using an equilibrium saturation method [27]. ITN was added in excess and combined with known quantities of each green solvent. The resulting mixture underwent a 10 min vortexing process. In a Water Shaker Bath (Daihan Scientific Co. Ltd., Seoul, Republic of Korea), the prepared samples were shaken at 100 rpm for 72 h [28,29]. After achieving equilibrium, each sample was collected, filtered, and centrifuged for 30 min at 5000 rpm. The supernatant was withdrawn carefully, diluted (when necessary), and used to determine ITN content in each sample by HPLC approach at 354 nm. The measured mole fraction solubility (*x*_e_) of ITN was obtained with the help of the following equation [25,26]:

(1) $$x_{e}=\frac{m_{1}/M_{1}}{m_{1}/M_{1}+m_{2}/M_{2}}$$

where *m*_1_ = ITN mass; *m*_2_ = green solvent mass; *M*_1_ = ITN molar mass; and *M*_2_ = green solvent molar mass. The solvent masses were taken on mass basis and calibration curve was also made on mass mass^−1^ basis. As a result, the density of solvents was not taken into consideration for the calculation of mole fraction solubility.

### 2.5. Determination of Solubility Parameters

Under normal conditions of temperature and pressure, the molecules with identical solubility characteristics may achieve the greatest solubility in the sample matrices [30]. Hence, this research assessed several solubility parameters for ITN and other environmentally friendly solvents. The overall “Hansen solubility parameter (HSP)” of ITN was calculated using the following equation [30,31,32]:

(2) $$\delta^{2}=\delta_{d}^{2}+\delta_{p}^{2}+\delta_{h}^{2}$$

where *δ* = total HSP of ITN; *δ*_d_ = dispersion HSP of ITN; *δ*_p_ = polar HSP of ITN; and *δ*_h_ = hydrogen-bonded HSP of ITN.

The values of *δ, δ*_d_, *δ*_p_, and *δ*_h_ for ITN were calculated by entering the simplified molecular input line entry system (SMILES) into the HSPiP program (version 4.1.07, Louisville, KY, USA). ITN’s PubChem database was used to obtain the SMLIES. The values of *δ, δ*_d_, *δ*_p_, and *δ*_h_ for other green solvents, on the other hand, were derived from reference [17].

The “van Krevelen and Hoftyzer solubility parameter (Δ$\;\bar{\delta }$) ” was calculated utilizing the following equation [33]:

(3) $$\Delta \;\bar{\delta }=\lceil \left(\delta_{d}{}_{2}^{2}-\delta_{d}{}_{1}^{2}\right)+\left(\delta_{p}{}_{2}^{2}-\delta_{p}{}_{1}^{2}\right)+{\left(\delta_{h}{}_{2}^{2}-\delta_{h}{}_{1}^{2}\right)\rceil }^{1/2}$$

ITN and a particular green solvent are denoted by subscripts 1 and 2, respectively. According to a prior publication, the molecule will be most soluble in the green solvent if Δ$\;\bar{\delta }$ < 5.0 MPa^1/2^ [33,34].

The “three dimensional (3D) solubility parameter space (*R*_a_)” was determined with the help of the following equation [35,36]:

(4) $$R_{a}{}^{2}=4{\left(\delta_{d2}-\delta_{d1}\right)}^{2}+{\left(\delta_{p2}-\delta_{p1}\right)}^{2}+{\left(\delta_{h2}-\delta_{h1}\right)}^{2}$$

According to a prior publication, the molecule will be most soluble in the green solvent if *R*_a_ < 5.6 MPa^1/2^ [35].

The HSP sphere for ITN using eleven distinct green solvents was also determined using HSPiP program. The HSP sphere or interaction radius (*R*_0_) was obtained using HSP sphere [37,38]. The relative energy difference (*RED*) was calculated with the help of HSP sphere using the following equation [39,40]:

(5) $$RED=\frac{R_{a}}{R_{0}}$$

The “Greenhalgh’s solubility parameter (∆*δ*)” was determined with the help of the following equation [41]:

(6) $$\Delta \delta =\delta_{2}-\delta_{1}$$

According to a prior publication, the molecule will be most soluble in the green solvent if ∆*δ* < 7.0 MPa^1/2^ [31,41].

### 2.6. Ideal Solubility of ITN and Solute–Solvent Interactions

The purpose of calculating an ideal solubility (*x*^idl^) was to determine activity coefficients of ITN, which were further used to describe solute–solvent interactions. The *x*^idl^ of ITN at different temperatures was determined with the help of the following equation [42]:

(7) $$\ln\;x^{\mathrm{idl}}=\frac{-\Delta H_{\mathrm{fus}}\left(T_{\mathrm{fus}}-T\right)}{RT_{\mathrm{fus}}T}+\left(\frac{\Delta C_{p}}{R}\right)[\frac{T_{\mathrm{fus}}-T}{T}+\ln\left(\frac{T}{T_{\mathrm{fus}}}\right)]\;$$

where ∆*C*_p_ is the difference between the molecular heat capacities of the solid phase and liquid state of ITN; *T*_fus_ is the ITN fusion temperature; ∆*H*_fus_ is the ITN fusion enthalpy; and *R* is the universal gas constant. The ∆*C*_p_ for ITN was calculated with the help of the following equation [42,43]:

(8) $$\Delta C_{p}=\frac{\Delta H_{\mathrm{fus}}}{T_{\mathrm{fus}}}$$

The *T*_fus_ and ∆*H*_fus_ for ITN were determined as 452.7 K and 7.64 kJ mol^−1^, respectively, by DSC analysis. Utilizing Equation (7), the ∆*C*_p_ for ITN was calculated as 16.67 J mol^−1^ K^−1^. Now, utilizing Equation (6), the *x*^idl^ data for ITN were derived.

The “activity coefficients (*γ*_i_)” for ITN in eleven different green solvents at different temperatures were determined with the help of the following equation [42,44]:

(9) $$\gamma_{i}=\frac{x^{\mathrm{idl}}}{x_{e}}$$

Based on the recorded *γ*_i_ values of ITN at different temperatures, ITN–solvent molecular interactions were determined.

### 2.7. ITN Solubility Correlation Using Computational Models

Computational modeling of the measured solubility of ITN is essential for practical validations [45,46]. In order to connect the measured ITN solubility data, “Apelblat, van’t Hoff, and Buchowski–Ksiazczak *λh* models” were used [31,47,48,49,50,51]. The “Apelblat model solubility (*x*^Apl^)” of ITN was determined with the help of the following equation [47,48]:

(10) $$\ln\;x^{\mathrm{Apl}}=A+\frac{B}{T}+C\ln\left(T\right)$$

where *A, B,* and *C* are the model coefficients derived from the measured ITN solubility values listed in Table 1, utilizing multivariate regression analysis [31]. Root mean square deviation (*RMSD*) and *R*^2^ were used to correlate *x*_e_ and *x*^Apl^ values of ITN. The *RMSD* of ITN was calculated utilizing its reported formula [18].

The “van’t Hoff model solubility (*x*^van’t^)” of ITN was determined with the help of the following equation [31]:

(11) $$\ln\;x^{\mathrm{van}\prime t}=a+\frac{b}{T}$$

where *a* and *b* are the “van’t Hoff model” coefficients that were established using the least squares method [52].

The “Buchowski–Ksiazczak *λh* solubility (*x*^λh^)” for ITN was determined with the help of the following equation [49,50]:

(12) $$\ln\;[1+\frac{\lambda (1-x^{\lambda h})}{x^{\lambda h}}]=\lambda h\;[\frac{1}{T}-\frac{1}{T_{\mathrm{fus}}}]$$

where *λ* and *h* are the model coefficients of “Buchowski–Ksiazczak *λh* model”.

### 2.8. Thermodynamic Analysis

Apparent thermodynamic analysis was applied to estimate the thermodynamic properties of ITN in eleven different green solvents. The “van’t Hoff and Krug et al. analysis” was used to identify three different parameters for ITN [44,53,54]. The apparent standard enthalpy (Δ_sol_*H*^0^) for ITN in eleven different green solvents was determined at the mean harmonic temperature (*T*_hm_) of 308 K using “van’t Hoff analysis” with the help of the following equation [44,53]:

(13) ∂ln xe∂1T−1ThmP=−ΔsolH0R

**Figure 2:**
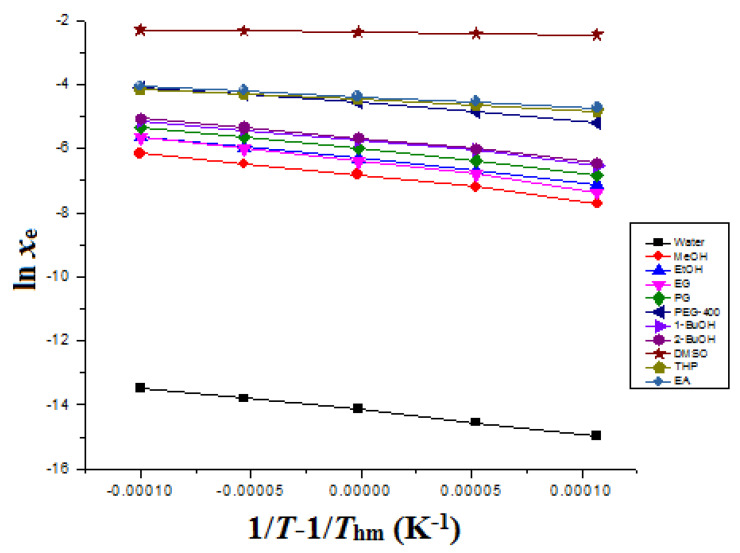
van’t Hoff curves for ITN in eleven distinct green solvents.

The Δ_sol_*H*^0^ for ITN was obtained from “van’t Hoff” graphs plotted between ln *x*_e_ values of ITN and 1T−1Thm. The van’t Hoff graphs for ITN in eleven different green solvents are mentioned in Figure 2. The Δ_sol_*H*^0^ for ITN was determined at *T*_hm_ according to “van’t Hoff analysis” (Equation (13)) instead of the standard temperature of 298.2 K [44,53].

Additionally, at *T*_hm_ = 308 K, the apparent standard Gibbs energy (Δ_sol_*G*^0^) for ITN in eleven different green solvents was also estimated using the “Krug et al. analysis” with the help of the following Equation (14) [53]:

(14) $$\Delta_{\mathrm{sol}}G^{0}=-RT_{\mathrm{hm}}\times \mathrm{intercept}$$

where the intercept values for ITN in eleven different green solvents were obtained from “van’t Hoff plots” mentioned in Figure 2.

The apparent standard entropy (Δ_sol_*S*^0^) for ITN dissolution was determined with the help of the following equation [44,53,54]:

(15) $$\Delta_{\mathrm{sol}}S^{0}=\frac{\Delta_{\mathrm{sol}}H^{0}-\Delta_{\mathrm{sol}}G^{0}}{T_{\mathrm{hm}}}$$

### 2.9. Statistical Analysis

The Kruskal–Wallis test was utilized, followed by Denn’s test for statistical comparison. GraphpadInstat software (San Diego, CA, USA) was used for this analysis. The *p* < 0.05 was considered a significant value.

## 3. Results and Discussion

### 3.1. Characterization of Solid Phases of ITN

**Figure 3:**
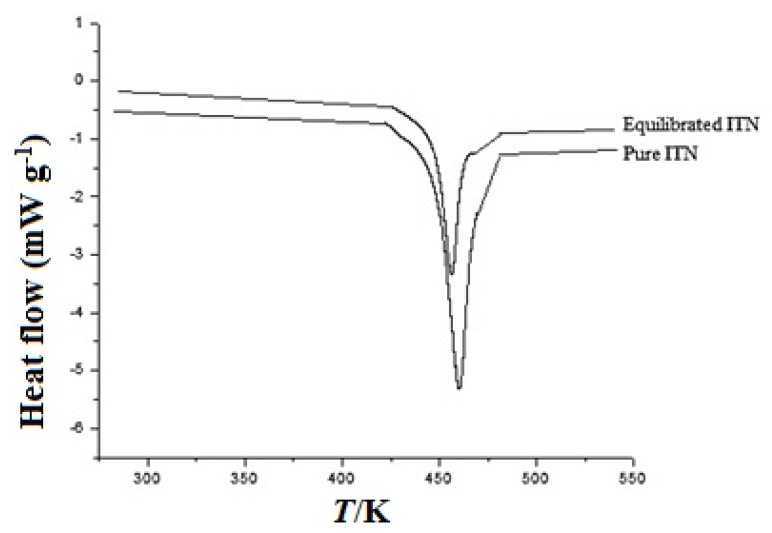
Differential scanning calorimetry (DSC) spectrum of pure and equilibrated ITN recovered from MeOH.

Pure and equilibrated ITN recovered from MeOH was used to characterize the solid phases of ITN using DSC, FTIR, and XRD spectrum analyses. Figure 3 displays the DSC spectrum of ITN in the pure and equilibrated samples. The endothermic peak of pure ITN in the DSC spectra, which corresponds to the *T*_fus_ value of ITN, was visible at 452.70 K (Figure 2). It was discovered that the pure ITN’s ∆*H*_fus_ value was 7.64 kJ mol^−1^. The endothermic peak of equilibrated ITN at 450.2 K, which denotes the *T*_fus_ of equilibrated ITN, was also seen in the DSC spectra of equilibrated ITN (recovered from MeOH). It was discovered that the equilibrated ITT’s ∆*H*_fus_ was 8.18 kJ mol^−1^. Pure and equilibrated ITN were found to have identical DSC spectra and thermal characteristics (*p* > 0.05). The *T*_fus_ of ITN was reported in the range of 450.20–457.82 K [1,2,3,19,23]. The *T*_fus_ of ITN was recorded to be 450.20 K by Ghorab and Babiker [3]. Berbenni et al. obtained the *T*_fus_ of ITN as 453.20 K [1]. Guimaraes et al. reported the *T*_fus_ of ITN as 454.29 K [19]. However, Ascenso et al. and Chavda et al. reported the *T*_fus_ of ITN as 457.30 K and 457.82 K, respectively [2,23]. ITN’s measured *T*_fus_ value at 452.70 K matched that found in the literature [1,2,3,19,23].

**Figure 4:**
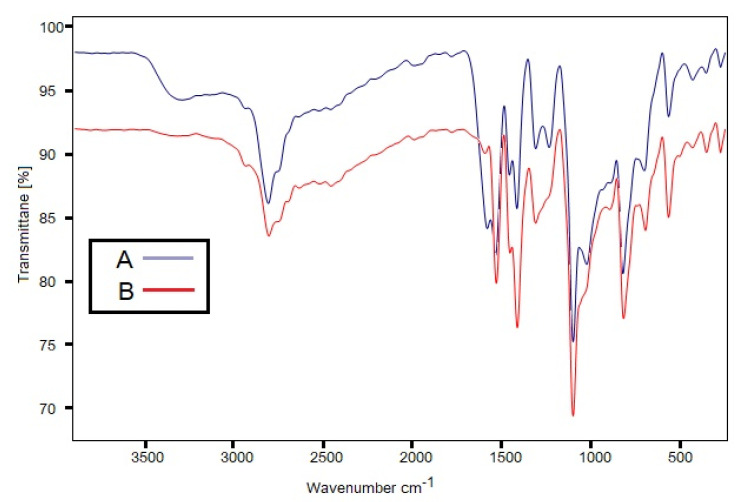
Fourier transform infrared (FTIR) spectrum of (**A**) pure ITN and (**B**) equilibrated ITN obtained from MeOH.

**Figure 5:**
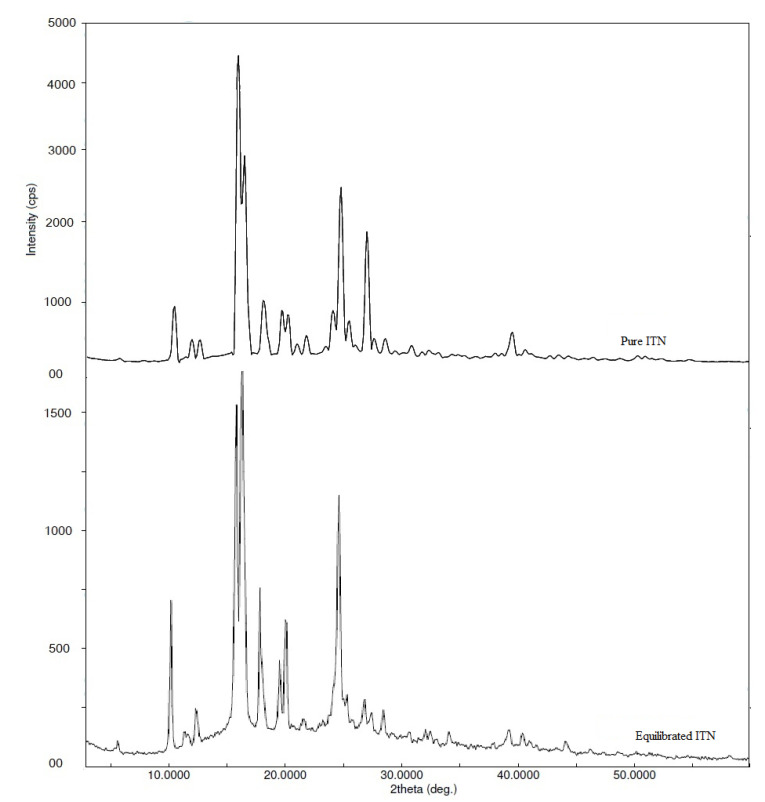
X-ray diffraction (XRD) patterns of pure and equilibrated ITN obtained from MeOH.

The FTIR spectra of pure and equilibrated ITN are shown in Figure 4. The crystalline nature of pure ITN was revealed by the distinct ITN characteristic peaks in the FTIR spectra of pure ITN at various wave numbers (Figure 4). Equilibrated ITN’s FTIR spectra showed identical ITN characteristics peaks at various wave numbers after being recovered from MeOH (Figure 4), proving that it is also crystalline. The XRD spectra of pure and equilibrated ITN are shown in Figure 5. Pure ITN’s XRD spectrum revealed a number of characteristic peaks of ITN at various 2θ values, proving that it is crystallized (Figure 5). The XRD curve of equilibrated ITN obtained from MeOH displayed similar feature peaks of ITN at varied 2θ values, indicating that the substance is crystalline (Figure 5). ITN was not transformed into solvates, polymorphs, or hydrates after equilibrium, according to the results of the DSC, FTIR, and XRD analyses. Given that the experimental settings for other green solvents were comparable, it was also anticipated that ITN would retain its crystalline form in other solvents as well.

### 3.2. Measured Solubility of ITN

Table 1 contains the solubility data for ITN in eleven different environmentally friendly solvents at various temperatures and constant air pressure. ITN’s equilibrium solubility in various environmentally friendly solvents, such as PG, PEG-400, and THP, is documented [20,23]. However, it has not yet been possible to measure the temperature-dependent solubility data of ITN in water, MeOH, EtOH, 1-BuOH, 2-BuOH, EG, PG, PEG-400, THP, EA, and DMSO. According to Patel et al. [20], the equilibrium solubility of ITN in PG at 298.2 K is 4.11 mg g^−1^ (converted to 1.04 × 10^−3^ in mole fraction). In this work, the mole fraction solubility of ITN in PG at 298.2 K was calculated to be 1.07 × 10^−3^. According to Patel et al. [20], the equilibrium solubility of ITN in THP at 298.2 K is 18.00 mg g^−1^ or 7.97 × 10^−3^ in mole fraction. In addition, Chavda et al. [23] observed that the equilibrium solubility of ITN in THP at 298.2 K was 52.3 mg mL^−1^ (equivalent to 2.21 × 10^−2^ in mole fraction). The mole fraction solubility of ITN in THP was calculated in this paper to be 8.06 × 10^−3^ at 298.2 K. According to Chavda et al. [23], the equilibrium solubility of ITN in PEG-400 at 298.2 K is 12.2 mg mL^−1^ (translated to 1.78 × 10^−2^ in mole fraction). In this work, the mole fraction solubility of ITN in PEG-400 at 298.2 K was calculated to be 5.56 × 10^−3^. The solubility of ITN in PG and THP that was observed was in agreement with that reported by Patel et al. [20]. The solubility of ITN in THP and PEG-400, however, differed significantly from that which Chavda et al. [23] observed. These variations could be explained by variations in experimental parameters such as shaking frequency, equilibrium duration, and ITN measurement analytical technique [1,9]. The shaking frequency of the solubility measurement could influence the equilibrium time and hence the solubility of the drug.

The results shown in Table 1 are in good accord with other studies [28,29] in that ITN’s solubility significantly increased with the rise in temperature in all the green solvents evaluated (*p* < 0.05) [28,29]. At 318.2 K, the sequence of ITN solubility in eleven distinct green solvents was as follows: DMSO (1.01 × 10^−1^) > EA (1.73 × 10^−2^) > PEG-400 (1.66 × 10^−2^) > THP (1.59 × 10^−2^) > 2-BuOH (6.32 × 10^−3^) > 1-BuOH (5.88 × 10^−3^) > PG (4.83 × 10^−3^) > EtOH (3.51 × 10^−3^) > EG (3.49 × 10^−3^) > MeOH (2.10 × 10^−3^) > water (1.38 × 10^−6^). The mole fraction solubility of ITN was higher in nonpolar green solvents, such as DMSO, EA, PEG-400, THP, 2-BuOH, 1-BuOH, PG, EtOH, EG, and MeOH as compared to water. This was probably because ITN had a –COOH group, which could have strong molecular solvation with nonpolar green solvents. The ITN solubility in 1-BuOH and 2-BuOH was not considerably different because of the similar molecular structures and molar masses of 1-BuOH and 2-BuOH (*p* > 0.05). Furthermore, the polarity of 1-BuOH and 2-BuOH is also not considerably different (*p* > 0.05). The solubility of ITN in PG was slightly higher than EG because the polarity of PG is slightly lower than EG (*p* > 0.05). The solubility of ITN in EtOH was slightly higher than MeOH because the polarity of EtOH is slightly lower than MeOH (*p* > 0.05). However, the solubility of ITN in PEG-400, THP, EA, and DMSO was considerably higher than its solubility in other green solvents examined (*p* < 0.05). This was probably because PEG-400, THP, EA, and DMSO have lower polarity compared to the other green solvents examined [55]. DMSO may be the best green solvent for ITN solubilization because it had the highest ITN solubility among the other investigated green solvents.

### 3.3. Determination of HSPs

**Figure 6:**
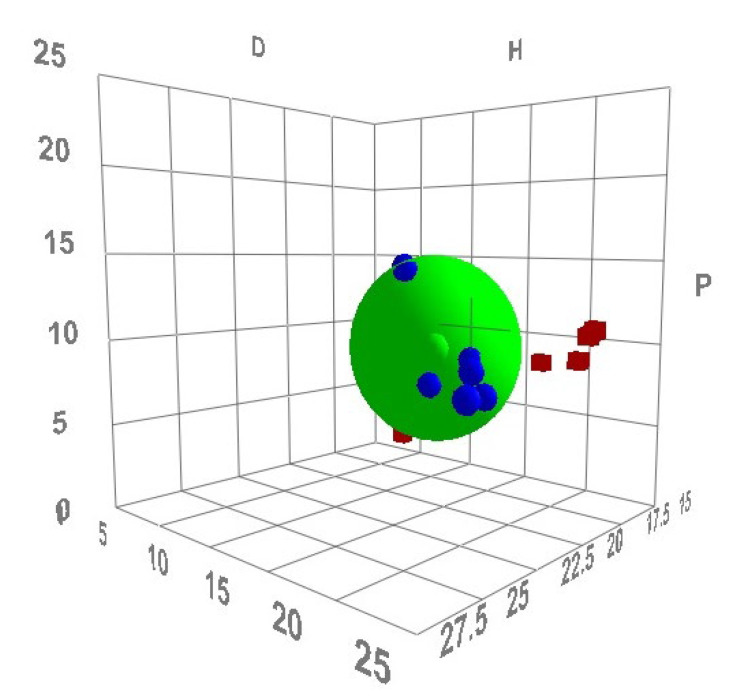
HSP sphere generated using HSPiP program.

“HSPiP software” was used to compute a number of HSPs for ITN. We used reference [17] to obtain the HSPs of eleven distinct environmentally friendly solvents. In Table 2, the results of the HSPs are listed. With a value *δ* value of 19.30 MPa^1/2^, ITN was discovered to have a low polarity. Six environmentally friendly solvents were identified as having identical *δ* values and being dependable for ITN solubilization: 1-BuOH, 2-BuOH, EA, DMSO, THP, and PEG-400 (Table 2). Water did not appear to be acceptable for ITN solubilization with a *δ* value of 47.80 MPa^1/2^. It was discovered that the solute will be most soluble in the green solvent if Δ$\;\bar{\delta }$ is < 5.0 MPa^1/2^ [33,34]. The Δ$\;\bar{\delta }$ value for four environmentally friendly solvents, including 2-BuOH (Δ$\;\bar{\delta }$ = 5.92 MPa^1/2^), THP (Δ$\;\bar{\delta }$ = 6.49 MPa^1/2^), PEG-400 (Δ$\;\bar{\delta }$ = 6.05 MPa^1/2^), and EA (Δ$\;\bar{\delta }$ = 3.58 MPa^1/2^), was close to the reported value, suggesting that these environmentally friendly solvents are appropriate for ITN solubilization, in accordance with this theory. It was also discovered that the solute will be most soluble in the green solvent if *R*_a_ is <5.6 MPa^1/2^ [35,36]. The *R*_a_ value for three environmentally friendly solvents, such as 2-BuOH (*R*_a_ = 6.69 MPa^1/2^), THP (*R*_a_ = 6.87 MPa^1/2^), and EA (*R*_a_ = 4.86 MPa^1/2^), was close to the reported value, suggesting that these environmentally friendly solvents are appropriate for ITN solubilization, in accordance with this theory. The 3D diagram for the HSP sphere generated using the HSPiP program is presented in Figure 6. The green solvents enclosed within the sphere are considered good solvents and the green solvents that are outside of the sphere are considered bad solvents. Using the values of *R*_a_ and *R*_0_, the *RED* for eleven distinct green solvents was determined and the data are presented in Table 2. The green solvents with a *RED* < 1.00 are considered good solvents. However, green solvents with a *RED* > 1.00 are considered bad solvents [39,40]. According to the HSP sphere and recorded *RED* values, the green solvents of 1-BuOH 2-BuOH, DMSO, THP, PEG-400, and EA were considered good solvents. However, the green solvents of water, MeOH, EtOH, EG, and PG were considered bad solvents. According to Greenhalgh’s principle, if ∆*δ* is < 7.0 MPa^1/2^, the solubility of the solute in the specific solvent will be higher. The insolubility of the solute is indicated by the value of ∆*δ* > 10.0 MPa^1/2^ [41]. Water had the greatest Δ*δ* value (∆*δ* = 28.50 MPa^1/2^), suggesting that the ITN was completely insoluble in it. However, the Δ*δ* value was determined to be within the specified range in EtOH (∆*δ* = 6.10 MPa^1/2^), 1-BuOH (Δ*δ* = 3.60 MPa^1/2^), 2-BuOH (Δ*δ* = 3.50 MPa^1/2^), DMSO (Δ*δ* = 4.30 MPa^1/2^), THP (Δ*δ* = 2.10 MPa^1/2^), PEG-400 (Δ*δ* = 0.40 MPa^1/2^), and EA (Δ*δ* = 1.20 MPa^1/2^), suggesting that ITN is completely soluble in all these green solvents, in accord with this principle [41].

### 3.4. Assessment of Solute–Solvent Interactions

Table 1 shows the *x*^idl^ data for ITN at five distinct temperatures. At 298.2–318.2 K, the *x*^idl^ values for ITN were recorded as 4.28 × 10^−1^ to 4.88 × 10^−2^. For ITN, it was discovered that the *x*^idl^ values were considerably higher than the *x*_e_ values in water (*p* < 0.05). On the other hand, it was discovered that ITN’s *x*^idl^ values were rather near to its *x*_e_ values in DMSO. These results demonstrated that DMSO was suitable for ITN solubility. Table 3 provides the *γ*_i_ values for ITN in eleven distinct green solvents at various temperatures. The *γ*_i_ values for ITN were discovered to be significantly higher in water compared to the green solvents. The *γ*_i_ values of ITN in eleven different green solvents considerably decreased with an increase in temperature (*p* < 0.05). The lowest *γ*_i_ values for ITN were discovered in DMSO. Based on these outcomes, ITN–DMSO was found to have the highest solute–solvent interactions when compared to other ITN–solvent combinations.

### 3.5. ITN Solubility Correlation Using Computational Models

**Figure 7:**
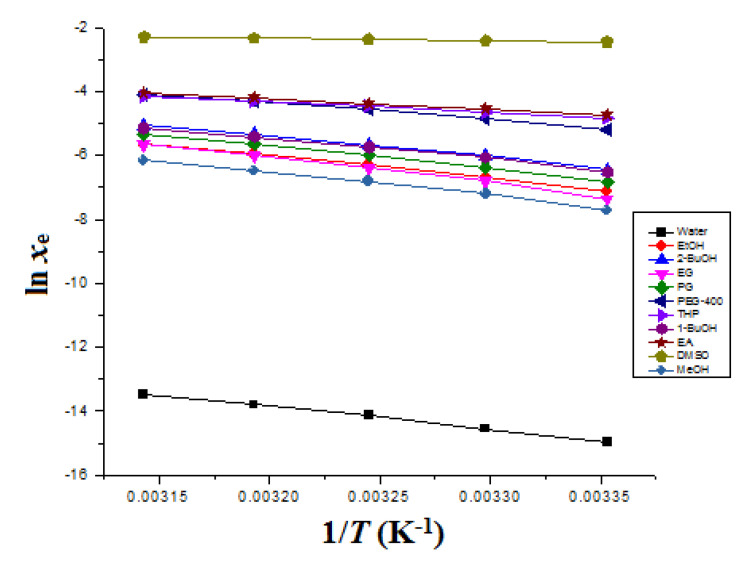
Experimental ITN solubility data in eleven distinct green solvents are correlated with the “Apelblat model” as a function of 1/T. Symbols indicate measured ITN solubility data, whereas solid lines indicate “Apelblat model” ITN solubility data.

The computational approaches used for correlation were the “van’t Hoff, Apelblat, and Buchowski–Ksiazaczak *λh* models” [31,47,48,49,50,51]. As a function of 1/*T*, Figure 7 displays the data for the graphical comparison between the *x*_e_ and *x*^Apl^ values of ITN in eleven distinct green solvents. This comparison indicated a high correlation between the *x*_e_ and *x*^Apl^ data of ITN in these eleven distinct green solvents. Table 4 provides an illustration of the results of the Apelblat model correlation. ITN’s overall *RMSD* was discovered to be 2.00%. *R*^2^ values ranged from 0.9984 to 0.9999 for ITN in eleven distinct environmentally friendly solvents. The observed solubility data for ITN in eleven distinct green solvents were found to be strongly correlated with the Apelblat model, as evidenced by the low *RMSD* and high *R*^2^ values.

The graph in Figure S1 compares the *x*_e_ and *x*^van’t^ values of ITN in eleven distinct green solvents as a function of 1/T and also shows a strong correlation between the two sets of data for ITN across all green solvents. Table 5 illustrates the findings of the “van’t Hoff model” analysis for ITN in eleven distinct environmentally friendly solvents. ITN was found to have an overall *RMSD* of 3.56% in eleven distinct green solvents. *R*^2^ values ranged from 0.9919 to 0.9999 for ITN in eleven distinct environmentally friendly solvents. The observed solubility data of ITN in eleven distinct green solvents were again shown to be well correlated with the “van’t Hoff model” by the low *RMSD* and high *R*^2^ values.

The results of the “Buchowski-Ksiazaczak *λh*” computation for ITN in eleven distinct environmentally friendly solvents are shown in Table 6. The overall *RMSD* for ITN was computed to be 4.41% in eleven distinct environmentally friendly solvents. The low *RMSD* values once more showed that the “Buchowski-Ksiazaczak *λh* model” and the measured solubility data of ITN in eleven distinct green solvents correlated very well. Finally, the solubility correlation of ITN showed good performance from all three computational approaches. 

### 3.6. Apparent Thermodynamic Analysis

The van’t Hoff plots, shown in Figure 2, were used to determine the Δ_sol_*H*^0^ values for ITN in eleven distinct environmentally friendly solvents. Table 7 presents the findings of an apparent thermodynamic analysis of ITN in eleven distinct environmentally friendly solvents. The ITN Δ_sol_*H*^0^ values were determined to be positive values between 6.382 and 66.90 kJ mol^−1^ in eleven distinct green solvents. It was also discovered that the ITX Δ_sol_*G*^0^ values in eleven distinct environmentally friendly solvents were positive and ranged from 6.071 to 36.33 kJ mol^−1^. As a result of the ITN measured solubility being maximum in DMSO and minimum in water, respectively, it was found that the ITN Δ_sol_*G*^0^ values were minimal in DMSO and greatest in water. ITN dissolution was endothermic in all of the green solvents tested, according to the positive values of Δ_sol_*H*^0^ for ITN [56,57]. ITN Δ_sol_*S*^0^ values were similarly found to be positive and ranged from 1.009 to 163.8 J mol^−1^ K^−1^ in eleven distinct green solvents, indicating an entropy-driven ITN dissolution across the board [56]. The ITN dissolution was proposed as an endothermic and entropy-driven process in all green solvents evaluated based on the positive values of Δ_sol_*H* and Δ_sol_*S*^0^ [56,57].

## 4. Conclusions

We reported on the solubility information, HSPs, and thermodynamic quantities of ITN in eleven distinct environmentally friendly solvents. The solid-state form of ITN was confirmed by the DSC, FTIR, and XRD investigations, which showed no change in ITN after equilibrium. The “van’t Hoff, Apelblat, and Buchowski-Ksiazczak *λh* models” found that the findings on ITN solubility were substantially associated. ITN became more soluble in each of the tested green solvents with an increase in temperature. ITN was soluble in eleven distinct environmentally friendly solvents in the following order: DMSO > EA > PEG-400 > THP > 2-BuOH > 1-BuOH > PG > EtOH > EG > MeOH > water at 318.2 K. The results of the activity coefficients showed that ITN–DMSO had the greatest molecular interactions when compared to other combinations of ITN and green solvents. Analyses of the apparent thermodynamic data revealed an “endothermic and entropy-driven dissolution” of ITN in all the green solvents tested. DMSO is recommended as the best green solvent for the solubilization of ITN based on all these results and discoveries. As a result, pre-formulation research and the design of ITN dosage forms can use DMSO as a viable green solvent.

## Figures and Tables

**Table 1:** Measured solubility (*x*_e_) and ideal solubility (*x*^idl^) data of isotretinoin (ITN) in mole fraction in eleven different green solvents (GS) at 298.2–318.2 K and 101.1 kPa ^a^.

GS	*x*_e_				
	*T* = 298.2 K	*T* = 303.2 K	*T* = 308.2 K	*T* = 313.2 K	*T* = 318.2 K
Water	3.16 × 10^−7^	4.80 × 10^−7^	7.20 × 10^−7^	1.02 × 10^−6^	1.38 × 10^−6^
MeOH	4.45 × 10^−4^	7.45 × 10^−4^	1.09 × 10^−3^	1.54 × 10^−3^	2.10 × 10^−3^
EG	6.27 × 10^−4^	1.11 × 10^−3^	1.65 × 10^−3^	2.47 × 10^−3^	3.49 × 10^−3^
EtOH	8.06 × 10^−4^	1.24 × 10^−3^	1.83 × 10^−3^	2.59 × 10^−3^	3.51 × 10^−3^
PG	1.07 × 10^−3^	1.71 × 10^−3^	2.52 × 10^−3^	3.53 × 10^−3^	4.88 × 10^−3^
1-BuOH	1.46 × 10^−3^	2.33 × 10^−3^	3.19 × 10^−3^	4.42 × 10^−3^	5.88 × 10^−3^
2-BuOH	1.56 × 10^−3^	2.46 × 10^−3^	3.44 × 10^−3^	4.83 × 10^−3^	6.32 × 10^−3^
PEG-400	5.56 × 10^−3^	7.92 × 10^−3^	1.06 × 10^−2^	1.33 × 10^−2^	1.66 × 10^−2^
THP	8.06 × 10^−3^	9.64 × 10^−3^	1.14 × 10^−2^	1.36 × 10^−2^	1.59 × 10^−2^
EA	8.82 × 10^−3^	1.07 × 10^−2^	1.25 × 10^−2^	1.52 × 10^−2^	1.73 × 10^−2^
DMSO	8.64 × 10^−2^	8.95 × 10^−2^	9.29 × 10^−2^	9.74 × 10^−2^	1.01 × 10^−1^
*x* ^idl^	4.28 × 10^−1^	4.43 × 10^−1^	4.58 × 10^−1^	4.73 × 10^−1^	4.88 × 10^−1^

^a^ The relative uncertainties *u*_r_ are *u*_r_(*T*) = 0.014, *u*_r_(*p*) = 0.003, and *u*_r_(*x*_e_) = 0.015.

**Table 2:** Several solubility parameters of ITN and eleven distinct green solvents at 298.2 K.

Components	HSPs				*R*_a_ */MPa^1/2^	∆*δδ*/MPa^1/2^	∆*δ* */MPa^1/2^	*RED* (*R*_a_*/R*_0_)
	*δ*_d_/MPa^1/2^	*δ*_p_/MPa^1/2^	*δ*_h_/MPa^1/2^	*δ*/MPa^1/2^				
ITN	17.60	2.60	7.50	19.30	-	-	-	-
Water	15.50	16.00	42.30	47.80	37.52	37.34	28.50	5.30
MeOH	17.40	10.60	22.40	30.30	16.91	16.91	11.00	1.94
EG	18.00	11.10	23.40	31.60	18.04	18.03	12.30	2.12
EtOH	16.20	8.40	17.60	25.40	11.97	11.73	6.10	1.23
PG	17.40	9.10	21.70	29.20	15.62	15.61	9.90	1.81
1-BuOH	15.90	6.30	15.20	22.90	9.19	8.71	3.60	0.97
2-BuOH	15.80	5.40	12.40	20.80	6.69	5.92	3.50	0.86
PEG-400	14.60	7.50	9.40	18.90	7.97	6.05	0.40	0.99
THP	16.30	7.20	11.90	21.40	6.87	6.49	2.10	0.54
EA	15.70	5.60	7.00	18.10	4.86	3.58	1.20	0.97
DMSO	17.40	14.20	7.30	23.60	11.60	11.60	4.30	0.99

* These numbers were computed between ITN and appropriate green solvent.

**Table 3:** Activity coefficients (*γ*_i_) of ITN in eleven different green solvents at 298.2–318.2 K.

GS	*γ*_i_				
	*T* = 298.2 K	*T* = 303.2 K	*T* = 308.2 K	*T* = 313.2 K	*T* = 318.2 K
Water	1,356,095	923,995.2	636,792.1	464,426.5	354,487.0
MeOH	961.7445	594.1927	417.5994	306.6269	232.0172
EG	682.7352	397.7430	277.6888	191.4309	139.6863
EtOH	531.2276	357.3747	249.5203	182.1199	139.1357
PG	399.4916	257.8128	181.3776	133.9927	101.0275
1-BuOH	291.8713	189.5621	143.3259	107.0819	83.05753
2-BuOH	273.9716	180.1061	133.1211	97.88820	77.29692
PEG-400	77.06326	55.92912	42.94635	35.33440	29.40221
THP	53.15250	45.97158	39.92065	34.66999	30.56384
EA	48.58303	41.29086	36.62448	31.10461	28.22605
DMSO	4.957833	4.952277	4.930169	4.860074	4.820541

**Table 4:** ITN Apelblat model results in terms of model parameters (*A, B* and *C*), *R*^2^, and root mean square deviation (*RMSD*) for eleven distinct green solvents.

GS	*A*	*B*	*C*	*R* ^2^	Overall *RMSD* (%)
Water	869.29	−46,552	−127.80	0.9996	
MeOH	1593.1	−79,662	−234.08	0.9992	
EG	1573.8	−79,404	−230.79	0.9990	
EtOH	956.05	−50,146	−139.53	0.9998	
PG	1209.3	−61,839	−177.05	0.9995	
1-BuOH	1229.9	−62,257	−180.37	0.9987	2.00
2-BuOH	1302.4	−65,672	−191.07	0.9997	
PEG-400	1196.9	−59,554	−175.93	0.9989	
THP	36.256	−4654.6	−4.4703	0.9999	
EA	304.76	−16,944	−44.348	0.9984	
DMSO	−112.62	4405.3	16.743	0.9985	

**Table 5:** ITN van’t Hoff model results in terms of model parameters (*a* and *b*), *R*^2^, and *RMSD* for eleven distinct green solvents.

GS	*a*	*b*	*R* ^2^	Overall *RMSD* (%)
Water	8.6318	−7030.1	0.9977	
MeOH	16.792	−7290.0	0.9929	
EG	19.677	−8047.2	0.9939	
EtOH	16.372	−6997.2	0.9975	
PG	17.000	−7094.9	0.9957	
1-BuOH	15.287	−6488.5	0.9939	3.56
2-BuOH	15.703	−6574.9	0.9945	
PEG-400	12.166	−5163.4	0.9919	
THP	6.1159	−3261.4	0.9999	
EA	6.0950	−3225.9	0.9972	
DMSO	0.11760	−766.58	0.9952	

**Table 6:** ITN “Buchowski-Ksiazaczak *λh* model” data for eleven distinct green solvents.

GS	*λ*	*h*	Overall *RMSD* (%)
Water	8.89740	1192.10	
MeOH	0.311000	23,440.5	
EG	0.900600	8935.37	
EtOH	0.084900	82,417.0	
PG	0.327900	21,637.3	
1-BuOH	0.045800	141,670	4.41
2-BuOH	0.134700	48,959.9	
PEG-400	0.239500	21,559.1	
THP	0.088400	36,893.6	
EA	0.030900	104,398	
DMSO	0.575800	1331.34	

**Table 7:** Apparent thermodynamic parameters (Δ_sol_*H*^0^, Δ_sol_*G*^0^, and Δ_sol_*S*^0^) along with *R*^2^ values for ITN in eleven distinct green solvents ^b^.

GS	Δ_sol_*H*^0^/kJ mol^−1^	Δ_sol_*G*^0^/kJ mol^−1^	Δ_sol_*S*^0^/J mol^−1^ K^−1^	*R* ^2^
Water	58.52	36.33	72.03	0.9976
MeOH	60.68	17.59	139.8	0.9927
EG	66.90	16.50	163.8	0.9937
EtOH	58.25	16.24	136.3	0.9974
PG	59.06	15.44	141.6	0.9966
1-BuOH	54.01	14.78	127.3	0.9937
2-BuOH	54.90	14.61	130.7	0.9944
PEG-400	42.98	11.76	101.3	0.9917
THP	27.15	11.44	50.97	0.9999
EA	26.85	11.20	50.79	0.9972
DMSO	6.382	6.071	1.009	0.9954

^b^ The relative uncertainties are *u*(Δ_sol_*H*^0^) = 0.040, *u*(Δ_sol_*G*^0^) = 0.048 and *u*(Δ_sol_*S*^0^) = 0.050.

## Data Availability

This study did not report any data.

## Supplementary Materials

The following supporting information can be downloaded at: https://www.mdpi.com/article/10.3390/ma15228274/s1, Figure S1: Correlation of measured solubility data of ITN with “van’t Hoff model” in eleven different green solvents as a function of 1/*T*; symbols represent the experimental solubility data of ITN and the solid lines represent the solubility data of ITN calculated by “van’t Hoff model”; Table S1: The specifications of materials.
