# Supplementary material for: Solubilization and Thermodynamic Analysis of Isotretinoin in Eleven Different Green Solvents at Different Temperatures
Source: Materials (Basel). 2022 Nov 21;15(22):8274. doi: 10.3390/ma15228274 (PMC9692401; doi:10.3390/ma15228274)
Supplement: Supplementary file 1 [file materials-15-08274-s001.zip › materials-1989035-supplementary.pdf]

<u>**Supporting information**</u>

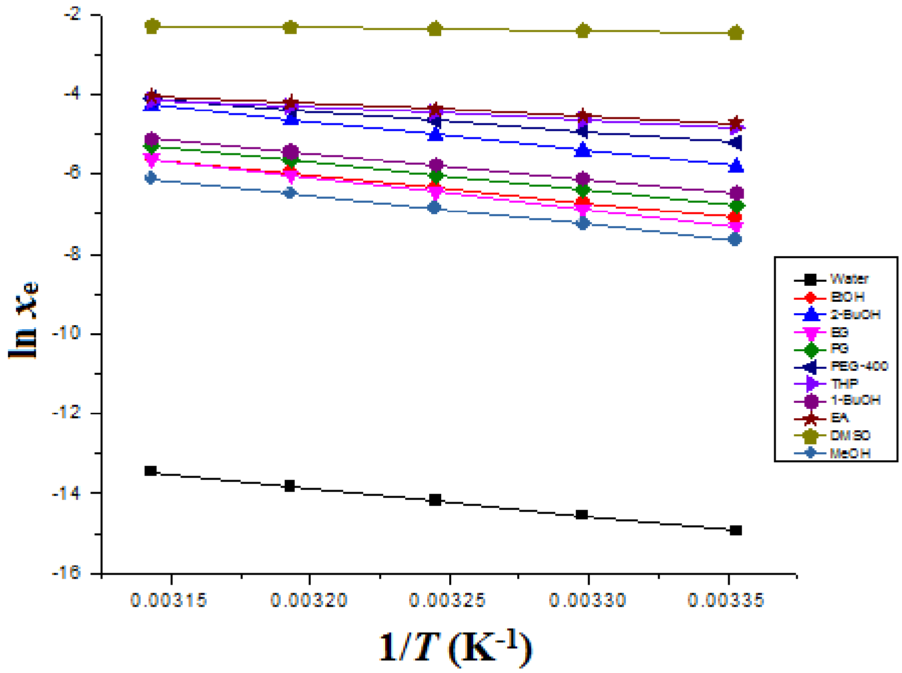

Figure S1. Correlation of measured solubility data of ITN with "van't Hoff model" in eleven different greener solvents as a function of 1/T; symbols represent the experimental solubility data of ITN and the solid lines represent the solubility data of ITN calculated by "van't Hoff model".

Table S1. The specifications of materials.

Material	Molecular formula	Molar mass (g mol^{-1})	CAS Registry no.	Purification method	Mass fraction purity	Analysis method	Source
ITN	$C_{20}H_{28}O_2$	300.40	4759-48-2	None	>0.98	HPLC	BOC Sciences
MeOH	CH_3OH	32.04	67-56-1	None	>0.99	GC	Fisher Chemical
EtOH	C_2H_5OH	46.07	64-17-5	None	>0.99	GC	Fisher Chemical
1-BuOH	$C_4H_{10}O$	74.12	71-36-3	None	>0.99	GC	Fisher Chemical
2-BuOH	$C_4H_{10}O$	74.12	78-92-2	None	>0.99	GC	Fisher Chemical
EG	$C_2H_6O_2$	62.07	107-21-1	None	>0.99	GC	Fisher Chemical
PG	$C_3H_8O_2$	76.09	57-55-6	None	>0.99	GC	Fisher Chemical
PEG-400	$H(OCH_2CH_2)_nOH$	400	25322-68-3	None	>0.99	HPLC	Fisher Chemical
THP	$C_6H_{14}O_3$	134.17	111-90-0	None	>0.99	GC	Gattefosse
DMSO	C_2H_6OS	78.13	67-68-5	None	>0.99	GC	Sigma Aldrich
EA	$C_4H_8O_2$	88.11	141-78-6	None	>0.99	GC	Sigma Aldrich
Water	H_2O	18.07	7732-18-5	None	-	-	Milli-Q

Both the analysis method and purity were provided by the supplier of each material.
